# The Acute Effects of the Use of Salbutamol and Ipratropium on the Heart Rates of Patients With Obstructive Airway Disease

**DOI:** 10.7759/cureus.46409

**Published:** 2023-10-03

**Authors:** Ayesha Akhtar, Syed Ali Abbas, Syed Haris M Zaidi, Adeel Sohail, Muhammad I Alam, Liaquat Raza

**Affiliations:** 1 Chest Medicine, Dr. Ziauddin University Hospital, Karachi, PAK; 2 Pulmonology and Critical Care, Dr. Ziauddin University Hospital, Karachi, PAK; 3 Internal Medicine, Dr. Ziauddin University Hospital, Karachi, PAK; 4 Critical Care Medicine, Dr. Ziauddin University Hospital, Karachi, PAK; 5 Medicine, Hamdard University, Karachi, PAK

**Keywords:** obstructive airway disease, cardiovascular effects, bronchodilator, ipratropium bromide, salbutamol

## Abstract

Background

The cornerstone of pharmaceutical therapy for obstructive airway illnesses involves inhalation of bronchodilators, such as ipratropium bromide (IP) and salbutamol (SB). The heart rate regulation may be changed by β-2 agonists and anticholinergic medications. Investigating the impact of inhaled SB and IP on the heart rate was the goal of this study.

Methods

A total of 304 patients were enrolled in this investigation. Baseline demographic characteristics, medical history, and adverse events were documented. Their heart rates were monitored before and after bronchodilator administration. SB and IP were selected based on historical usage. Blood pressure readings were also taken before and after each session.

Results

There was a significant increase in heart rates after SB from a mean of 106.69 to 117.20. Similarly, the heart rate of the patients in the IP group increased to a mean of 106.95 from 93.44, with a statistically significant p-value. Moreover, tremors were the most common adverse effect, accounting for 85.3% of the patients in the IP group and 75% in the SB group. In contrast, palpitation was more common in the SB group 25% vs. 14.7% with a significant p-value.

Conclusion

Frequently administered dosages of SB and IP caused a considerable increase in heart rates, as well as tremors and palpitation.

## Introduction

Chronic obstructive pulmonary disease (COPD) is a common, preventable, and manageable condition. Asthma, among the most common major non-communicable diseases, significantly reduces many people's quality of life. Asthma already affects 300 million individuals worldwide, and another hundred million individuals could contract it by 2025 [1]. Reactive airway illnesses, such as COPD, often require bronchodilator therapy for effective management. Among the medications commonly used are the β2-receptor agonist salbutamol (SB) and the anticholinergic ipratropium bromide (IP). Bronchodilator therapy is a cornerstone in treating COPD and asthma exacerbations [2]. The typical bronchodilator treatment regimen may include β-adrenergic and aerosolized or systemic medications, anticholinergic medications, theophylline, and/or corticosteroid therapy.

While these drugs have demonstrated local bronchodilating effects, there have been concerns about their potential adverse systemic effects, particularly in individuals with cardiac comorbidities [3]. The high frequency of cardiac comorbidity and mortality in individuals with COPD may worsen due to the adrenergic effects of regular frequent intake of short-acting bronchodilators in clinical settings [4]. Therefore, Australian and New Zealand guidelines recommend against using β2-agonists for an extended period of time [5]. The use of long-acting β2-agonists has been cautioned against due to potential adverse cardiac effects [5], and hence it is crucial to examine the cardiovascular implications of short-acting bronchodilators, such as SB and IP.

Therefore, considering that these medicines constitute a cornerstone of COPD exacerbation care, taking into account potential benefits and risks, there is a need to synthesize current data for their usage. IP and SB can have adverse systemic effects in addition to their local broncho-dilating effects. Although the overall mortality in asthma is low, it is on the rise [6]. There are several potential causes, such as the lack of availability of and access to appropriate healthcare services, physicians' inability to appreciate the seriousness of asthma episodes, adverse effects of the drugs, and the presence of underlying chronic illnesses [7]. The following characteristics of beta-2 agonists may provide a rationale for this rise in mortality: the pulse, systolic pressure, and muscle contractility all increase due to the inhaled beta-2 agonist, increasing the heart's need for oxygen [8]. The hearts' β-2 receptors [9] and β-1 receptors [10] both mediate these actions unselectively. Inhaled SB and its deleterious effects, such as increased pulse, tremors, and arrhythmias, have been associated with a higher risk of dying from asthma [11].

Following absorption into the bloodstream, SB shortens the duration of diastole, raising heart rates and causing myocardial oxygen demand [12]. Additionally, it acts on β-adrenergic receptors in the cardiac muscle, increasing sympathetic outflow [12]. According to Kallergis et al., who looked at the electrical changes in the heart muscle following SB administration, SB can also cause a number of abnormalities, including atrioventricular (AV) delays and reduced AV refractoriness [13]. Similar effects have been reported with the infusions of beta-2 agonists used in late pregnancy to avoid premature labor, which occasionally resulted in cardiac damage [14]. Additionally, complications from an inhaled beta-2 agonist have been documented, including myocardial infarction [15]. Another potential side effect of this medication is increased susceptibility to arrhythmias [16]. Researchers have examined changes in blood pressure (BP) and heart rate to gauge the cardiovascular effects of albuterol and IP [17]. However, these metrics might not be sensitive to the more subtle effects of these medications on the autonomic nervous system, which regulates the cardiovascular system [18].

Nevertheless, it is not thought that people with coronary artery disease, COPD, or asthma usually develop angina pectoris, which is made worse by inhaling SB [19]. Despite this, SB's increased oxygen demand in the myocardium may cause potentially dangerous silent ischemia [20]. In order to determine if inhaled SB and IP cause adverse effects on the heart rates of individuals with asthma or COPD, we conducted the current study.

## Materials and methods

Subjects 

The study was conducted as a prospective cohort study at Dr. Ziauddin University Hospital, Karachi, Pakistan, for a period of six months from January 01, 2023, till June 30, 2023, which included patients between the ages of 18 and 65 years, of any gender, with obstructive airway disease. Patients who met the inclusion criteria were enrolled in the study after obtaining ethical review committee approval from the Dr. Ziauddin University Hospital Ethical Review Committee.

Patient selection

In our study, we included patients aged between 18 and 65 years of any gender, who were diagnosed with obstructive airway disease (asthma or chronic obstructive pulmonary disease), presenting to Dr. Ziauddin University Hospital. Participants who provided informed consent were enrolled.

We excluded patients with thyroid problems, a history of cardiac arrhythmias, pacemakers, other significant cardiac comorbidities (e.g., coronary artery disease), and pregnant women. Patients with cancer, psychiatric illnesses, and chronic liver and kidney diseases were also excluded. Participants who consumed alcohol or beverages with caffeine within 24 hours before the study were not included. Patients who were unable to provide informed consent were also excluded.

Sample size calculation

The sample size was calculated based on the anticipated effect size, significance level (α), and power (1-β) using the following formula:

n=(d)22(Zα/2+Zβ)2⋅(σ)2

where n is the required sample size; /2Zα/2 and Zβ are the critical values corresponding to the desired level of significance and power, respectively; σ is the estimated standard deviation; and d is the desired effect size.

Study design

Following the recruitment of eligible participants, heart rate measurements were manually recorded, using a heart rate monitor, by a trained attending nurse or duty doctor. Heart rate data were collected after the patient was made to sit or lie for a minimum of 10 minutes. Subsequently, the heart rate was measured 10 minutes after nebulization with either IP (250 mcg) or SB (2.5 mg). The choice of medication was based on the patient's previous history of bronchodilator use. Patients who had been previously taking short-acting β-agonists (SABA) or long-acting β-agonists (LABA) were administered SB, while those previously on short-acting muscarinic antagonists (SAMA) or long-acting muscarinic antagonists (LAMA) received IP. Patients who were taking bronchodilators from both groups were given SB or IP alternatively. Blood pressure readings were taken by a qualified clinician from the left arm held at heart level using a sphygmomanometer before and after each nebulization session.

Statistical analysis 

All statistical analyses were performed using Statistical Package for the Social Sciences (SPSS) (version 26; IBM SPSS Statistics for Windows, Armonk, NY). Categorical variables were compared using Fisher's exact test or the chi-square test, depending on the context. The Kolmogorov-Smirnov test was employed to assess the normal distribution of continuous variables before applying Student’s t-test to compare them. A paired sample t-test was used to analyze the mean heart rate before and after administering the medication. A p-value of 0.05 or less was considered statistically significant.

## Results

A total of 304 patients were included in this prospective cohort study. Table 1 presents the baseline demographic characteristics of the patients in the two groups. The mean age of patients in the IP group was significantly higher than those in the SB group (59.69 ± 17.65 vs. 44.24 ± 19.59; P = <0.001). The distribution of genders differed significantly between the groups, with males being more common in the IP group and females in the SB group (P = <0.001). Common comorbid conditions observed were hypertension, followed by diabetes and ischemic heart disease. Comorbid conditions were more frequently seen in the IP group, with statistically significant p-values. The SB group had a higher proportion of patients with asthma (70.2% vs. 23.5%; P = <0.001), while the IP group had a higher prevalence of COPD (45.6% vs. 14.3%; P = <0.001).

**Table 1:** Baseline characteristics of patients between two groups SD, standard deviation; IP, ipratropium bromide; SB, salbutamol P < 0.05, significant

	Total Frequency (Percentage)	IP Frequency (Percentage)	SB Frequency (Percentage)	P-value
Age (Mean ± SD)		59.69 ± 17.65	44.24 ± 19.59	<0.001
Gender				
Male	150 (49.3)	88 (64.7)	62 (36.9)	<0.001
Female	154 (50.7)	48 (35.3)	106 (63.1)	
Diabetes Mellitus	92 (30.3)	46 (33.8)	46 (27.4)	0.138
Hypertension	94 (30.9)	60 (44.1)	34 (20.2)	<0.001
Ischemic heart disease	62 (20.4)	42 (30.9)	20 (11.9)	<0.001
Chronic liver disease	24 (7.9)	16 (11.8)	8 (4.8)	0.021
Malignancy	44 (14.5)	30 (22.1)	14 (8.3)	0.001
Other therapy given	54 (17.8)	30 (22.1)	24 (14.3)	0.054
Smoking	116 (38.2)	72 (52.9)	44 (26.2)	<0.001
Asthma	150 (49.3)	32 (23.5)	118 (70.2)	<0.001
Chronic obstructive pulmonary disease	86 (28.3)	62 (45.6)	24 (14.3)	<0.001
Bronchiectasis	64 (21.1)	42 (30.9)	22 (13.1)	<0.001
Biomass exposure	66 (21.7)	38 (27.9)	28 (16.7)	0.013
Inhalers taken in the last six hours	64 (21.1)	22 (16.2)	42 (25)	0.041
Past history of bronchodilators				
Short-acting β-agonist	138 (45.4)	56 (41.2)	82 (48.8)	0.112
Long-acting β-agonist	90 (29.6)	36 (26.5)	54 (32.1)	0.171
Short-acting muscarinic antagonist	66 (21.7)	36 (26.5)	30 (17.9)	0.048
Long-acting muscarinic antagonist	54 (17.8)	42 (30.9)	12 (7.1)	<0.001
Duration of bronchodilator use (years) (Mean ± SD)		7.68 ± 8.05	6.30 ± 6.66	0.105

Additionally, bronchiectasis and biomass exposure were more predominant in the IP group (P = <0.001 and P = 0.013, respectively). Patients in the IP group had a longer history of bronchodilator use (7.68 years) compared to those in the SB group (6.30 years), although the difference was not statistically significant (P = 0.105).

The effects of the medications on heart rate are presented in Table 2. Patients in the SB group had a significantly greater mean pulse before taking the drug compared to those in the IP group (106.69 ± 17.59 vs. 93.44 ± 17.04; P = <0.001). After administering SB, there was a significant increase in their heart rates to 117.20 ± 20.87 (P = <0.001). Similarly, although the baseline heart rate of patients in the IP group was within the normal range, it increased significantly to 106.94 ± 22.73 after receiving IP (P = <0.001).

**Table 2:** Effect of the drugs on the pulse before and after giving the drug SD, standard deviation P < 0.05, significant

Drug	Pulse Before Giving the Drug	Pulse After Giving the Drug	P-value
Salbutamol (Mean ± SD)	106.69 ± 17.59	117.20 ± 20.87	<0.001
Ipratropium bromide (Mean ± SD)	93.44 ± 17.04	106.94 ± 22.73	<0.001

Other adverse effects are listed in Table 3. Tremors were the most common adverse effect, observed in 85.3% of patients in the IP group and 75% in the SB group (P = 0.018). Palpitations were more common in the SB group (25% vs. 14.7%; P = 0.018).

**Table 3:** Other adverse effects of the drugs IP, ipratropium bromide; SB, salbutamol P < 0.05, significant

Other side effects	Total Frequency (Percentage)	IP Frequency (Percentage)	SB Frequency (Percentage)	P-value
Tremors	242 (79.6)	116 (85.3)	126 (75)	0.018
Palpitation	62 (20.4)	20 (14.7)	42 (25)	0.018

## Discussion

The current study aimed to investigate the cardiovascular effects of two commonly used bronchodilators, SB, and IP, in patients with obstructive airway disease. The findings revealed several important insights into the impact of these medications on heart rates and other adverse effects, which have significant implications for clinical practice.

When evaluating the cardiovascular effects of these medicines, prior research has typically concentrated on changes in pulse and blood pressure changes [21]. The main conclusion of this investigation is that IP and SB have considerable impacts on heart rates and induce significant extrapulmonary side effects. According to prior studies, SB does not substantially impact the circulatory or metabolic systems when used at the prescribed doses [22]. Here, we have demonstrated that both medications have comparable and significant effects on patients' heart rates during a severe attack of airway blockage. These results are at odds with those of Bremner et al., who found that SB and hexoprenaline differed little in extrapulmonary effects in doses that patients may take during a severe asthma attack [21]. The study results demonstrated that both SB and IP had notable effects on heart rate. SB administration led to a substantial increase in heart rate, which is consistent with previous research indicating its beta-adrenergic agonist properties [8]. Similarly, IP also caused a significant rise in heart rates, albeit starting from a normal baseline value. This suggests that both medications, despite their different mechanisms of action (β2-agonist for SB and anticholinergic for IP), may have important cardiovascular implications.

The present study investigated the cardiovascular effects of SB and IP in patients with obstructive airway disease. Our findings revealed a significant increase in heart rates after the inhalation of both medications, which has important clinical implications. These results are consistent with previous research on the cardiovascular impact of these bronchodilators, highlighting the need for careful consideration of their use in patients with underlying cardiac comorbidities.

One notable finding in our study was the dose-dependent effect of SB on heart rates. A previous investigation demonstrated that a dose of 1.5 mg of SB increased heart rates by 14 beats per minute in one case and an average of seven beats per minute in three patients after 200 μg of the medication [23]. Another study reported a rise in heart rates of 15-25 beats per minute in case 2 after inhalation of a total dose of 2.6 mg of SB and in two other patients who inhaled cumulative doses of 1.6 and 3.1 mg, with the heart rates returning to baseline after 40-60 minutes. These findings align with the known β-adrenergic receptor-mediated increase in heart rate associated with SB administration, and caution is warranted, especially in patients not continuously using drugs from the same class [24]. Additionally, the favorable chronotropic effect of SB may be amplified in hypoxic conditions, which should be taken into consideration when managing patients exposed to hypoxia [25].

Our results are comparable with other studies that reported a rise in heart rates following the inhalation of SB [26]. However, some investigations did not observe a discernible increase in heart rates after SB delivery [27]. These discrepancies may be attributed to differences in study populations, study designs, and methods used to measure heart rates.

In contrast to SB, IP has been suggested as a preferable aerosol therapy option due to its lack of association with an increase in heart rates [28]. Our study contradicts this finding, as IP led to a significant change in heart rates in patients with obstructive airway disease.

The parasympathetic regulation of the heart is of utmost importance, and disturbances in parasympathetic activity can lead to arrhythmias and cardiac mortality [29]. Patients with hypoxemic COPD and cardiopulmonary illness may have subclinical autonomic neuropathy and longer electrocardiographic corrected QT intervals, respectively, making them susceptible to the effects of IP inhalation on reducing parasympathetic tone and potentially increasing the risk of arrhythmias [30].

## Conclusions

This study provides valuable insights into the cardiovascular effects of two commonly used bronchodilators, SB, and IP, in patients with obstructive airway disease. The results highlight the need for careful consideration of potential adverse effects, particularly in patients with pre-existing cardiovascular comorbidities. Clinicians should weigh the benefits and risks of each medication based on individual patient characteristics to ensure safe and effective management of obstructive airway disease. Future research should continue to explore the cardiovascular effects of bronchodilators, aiming to improve treatment strategies and patient outcomes.

## Appendices

Implementing a randomized controlled trial design: To further validate the findings and minimize potential biases, future studies should adopt a randomized controlled trial design when investigating the cardiovascular effects of SB and IP.

Long-term follow-up: Long-term follow-up studies are needed to assess the sustained cardiovascular effects of these bronchodilators over extended periods, particularly in patients with COPD and asthma.

Assessing dose-dependent effects: Investigating the dose-dependent cardiovascular effects of SB and IP can provide valuable insights into the safety profiles of different dosages and aid in optimizing treatment regimens.

Evaluating specific patient populations: Conducting subgroup analyses based on specific patient characteristics, such as age, gender, and presence of cardiovascular comorbidities, can help identify vulnerable populations and tailor bronchodilator therapy accordingly.

Exploring alternative bronchodilator options: Given the potential adverse cardiovascular effects observed in this study, exploring alternative bronchodilator options with potentially safer cardiovascular profiles may be beneficial, especially in patients with significant cardiac comorbidities.

Enhancing adverse event monitoring: Healthcare providers should be vigilant in monitoring adverse events associated with bronchodilator therapy, particularly heart rate changes and tremors, and promptly address any concerning findings to ensure patient safety.

Educating patients and healthcare professionals: Raising awareness among patients and healthcare professionals about the potential cardiovascular risks of bronchodilator therapy can lead to more informed treatment decisions and improved patient outcomes.

Emphasizing individualized treatment plans: Tailoring bronchodilator therapy based on individual patient characteristics, including their respiratory condition, medical history, and cardiovascular risk profile, is crucial to optimize treatment efficacy while minimizing potential harm.

Conducting real-world studies: Real-world studies in diverse patient populations and healthcare settings can provide valuable data on the cardiovascular effects of bronchodilators under everyday clinical conditions, complementing findings from controlled clinical trials.
